# Supplementary material for: Novel Injectable Fluorescent Polymeric Nanocarriers for Intervertebral Disc Application
Source: J Funct Biomater. 2023 Jan 17;14(2):52. doi: 10.3390/jfb14020052 (PMC9961171; doi:10.3390/jfb14020052)
Supplement: Supplementary file 1 [file jfb-14-00052-s001.zip › jfb-2156687-supplementary.pdf]

Supporting Information

Novel Polymeric Injectable Fluorescent Drug Delivery Nanoparticles: A Proof-of-concept Study for Intervertebral Disc Application

Michael R. Arul[1], Changli Zhang[2], Ibtihal Alahmadi[3], Isaac L. Moss[1], Yeshavanth Kumar Banasavadi-Siddegowda[4], Svenja Illien-Junger[2*], Sama Abdulmalik1, Sangamesh G. Kumbar[1,3,5*]

[1] Department of Orthopedic Surgery, University of Connecticut Health, Farmington, CT, USA
[2] Department of Orthopedic Surgery, Emory University, Atlanta, GA, USA
[3]Department of Biomedical Engineering, University of Connecticut, Storrs, CT, USA
[4]Surgical Neurology Branch, National Institute of Neurological Disorders and Stroke, National Institutes of Health, Bethesda, MD, USA
[5]Department of Materials Science and Engineering, University of Connecticut, Storrs, CT, USA

*Corresponding Author
Sangamesh G. Kumbar, Ph.D.
Professor
Department of Orthopedic Surgery
Department of Biomedical Engineering
Department of Materials Science and Engineering
The University of Connecticut
Farmington, CT 06030-4037
Ph: (860) 679-3955
Fax: (860) 679-1474
Email: kumbar@uchc.edu

Svenja Illien-Junger, PhD
Assistant Professor
Department of Orthopaedics
Emory University
email: svenja.illien-junger@emory.edu

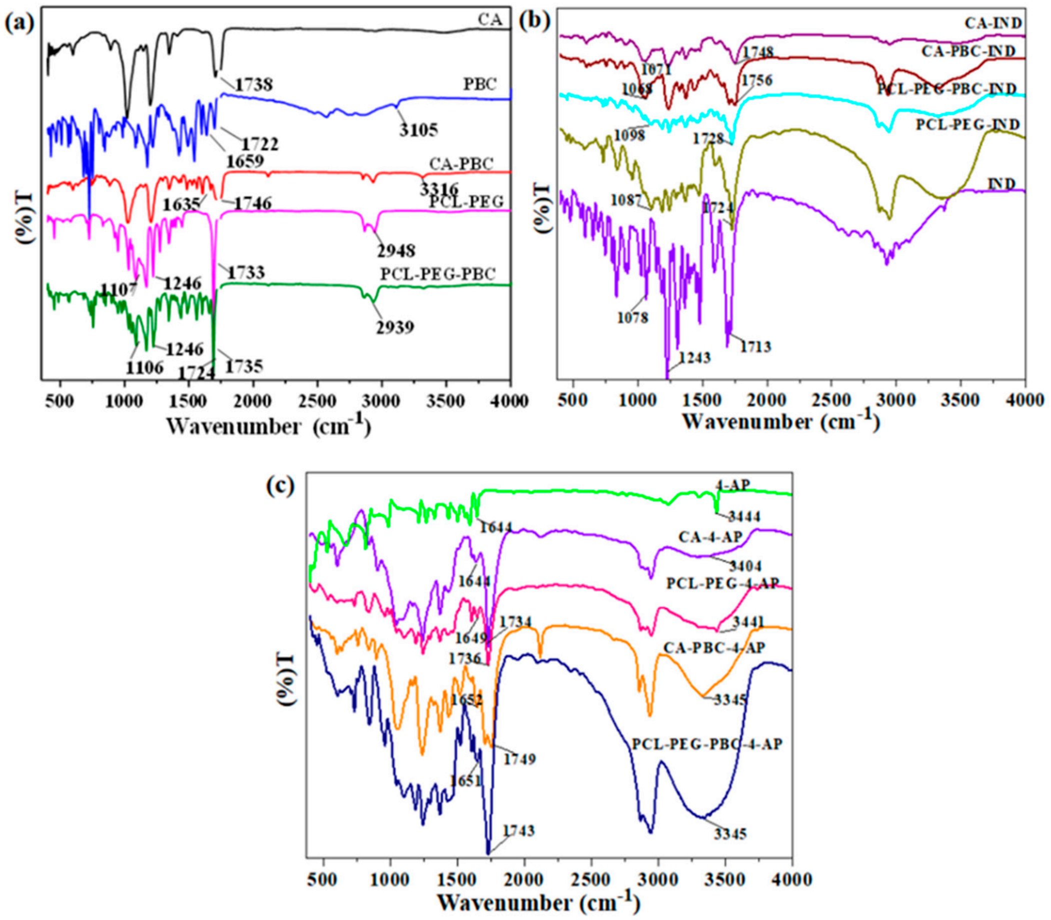

Figure S1. (a) FTIR spectroscopy of PBC, CA, CA-PBC, PCL-PEG, and PCL-PEG-PBC; (b) IND, CA-IND, CA-PBC-IND, PCL-PEG-IND, and PCL-PEG-PBC-IND; and (c) 4-AP, CA-4-AP, CA-PBC-4-AP, PCL-PEG-4-AP, and PCL-PEG-PBC-4-AP. CA, cellulose acetate; PCL, polycaprolactone; PEG, polyethylene glycol; 4-AP, 4-aminopyridine. Characteristic stretching PBC dye has characteristic bands at 1722 cm^{-1} for -C=O stretching and amide at 1659 cm^{-1} confirms the dye formation. The characteristic PBC-conjugated CA and PCL-PEG showed −C=O stretching shift from 1738 cm^{-1} to 1746 cm^{-1} and 3538 cm^{-1} confirming the formation of dye conjugation. The shifting of the distinct peaks of carbonyl (–C=O) stretching 1713 cm-1 of the IND to 1748 cm^{-1}, and 4-AP of NH_2 stretching 1644 cm^{-1} shifted to 1646 cm^{-1} indicates the drug interaction with nanoparticles.

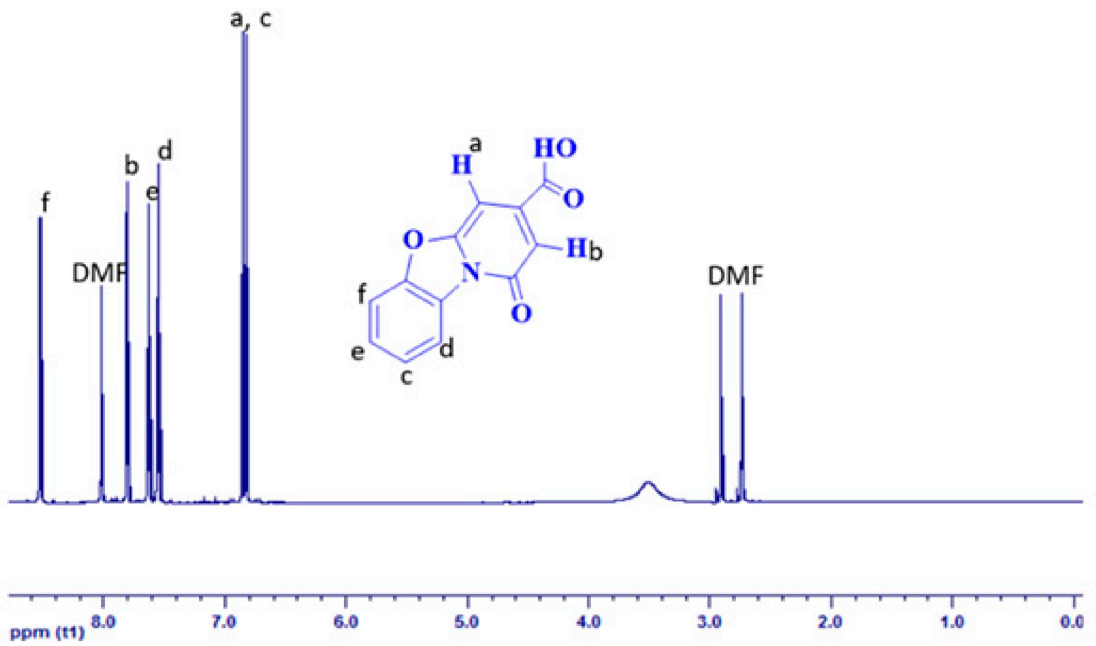

Figure S2. Structure characterization of synthesized copolymers by ^{1}H NMR for PBC dissolved in DMF as the solvent. The peaks of PBC at 8.01 and 6.82 ppm designated as a and b belong to -CH- of the 6-membered ring, and it confirms the incorporation of citric acid into the 2-aminophenol compound.

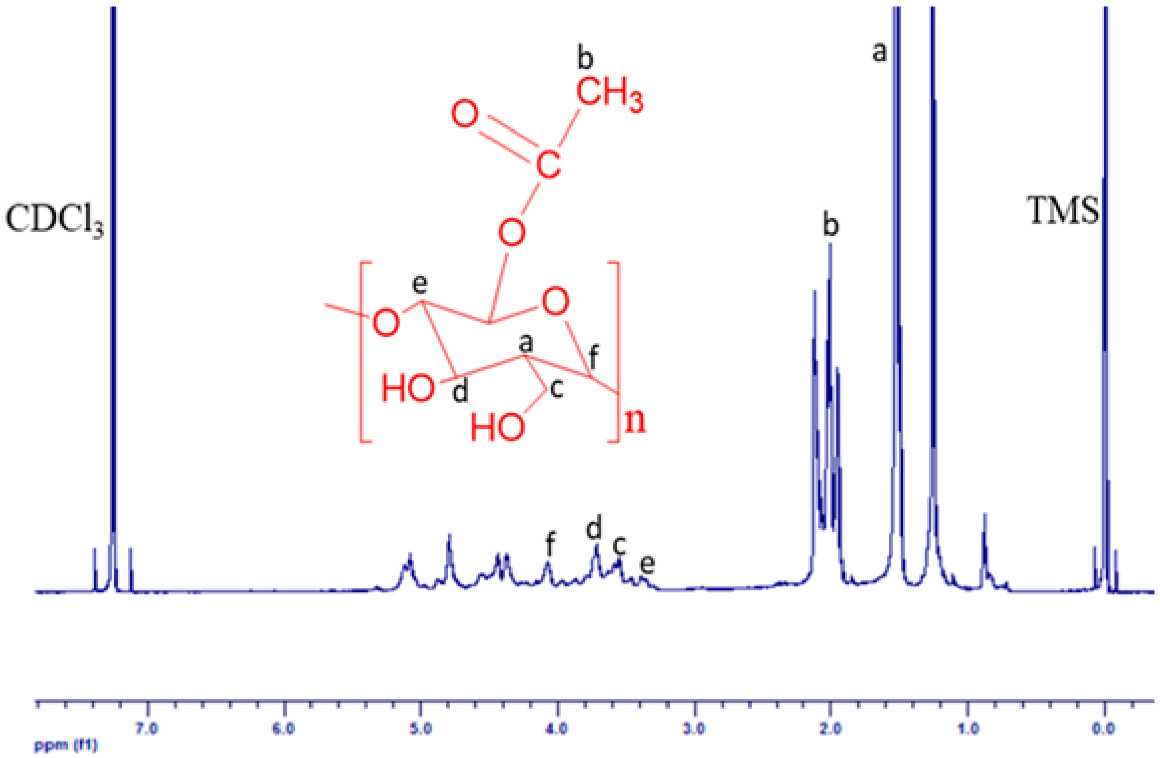

Figure S3. Structure characterization of synthesized copolymers by ^1H NMR for CA dissolved in $CDCl_3$ as the solvent.

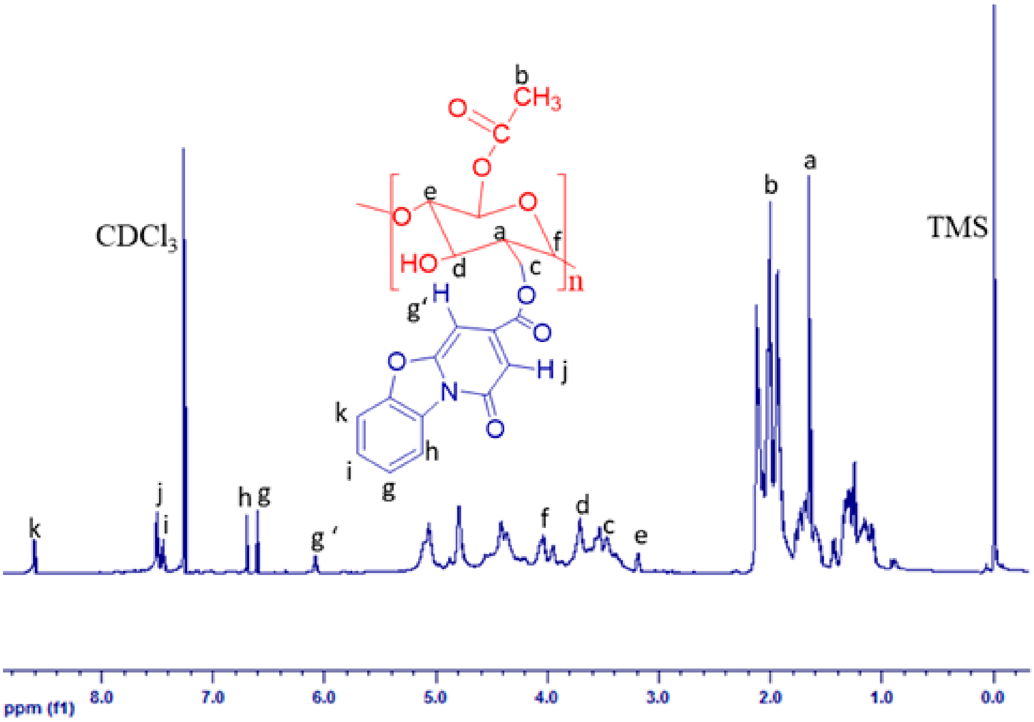

Figure S4. Structure characterization of synthesized copolymers by ^{1}H NMR for CA-PBC dissolved in $CDCl_3$ as the solvent. The peaks of PBC in d,e, and f with δ values at 3.37 ppm, 3.72 ppm, and 4.02 ppm are of the CA backbone confirming the formation of CA-PBC. Also, the appearance of the PBC benzene ring at δ values of 6.61 ppm, 6.70 ppm, 7.42 ppm, and 8.63 ppm (g,h, i,k) confirms PBC conjugation to CA.

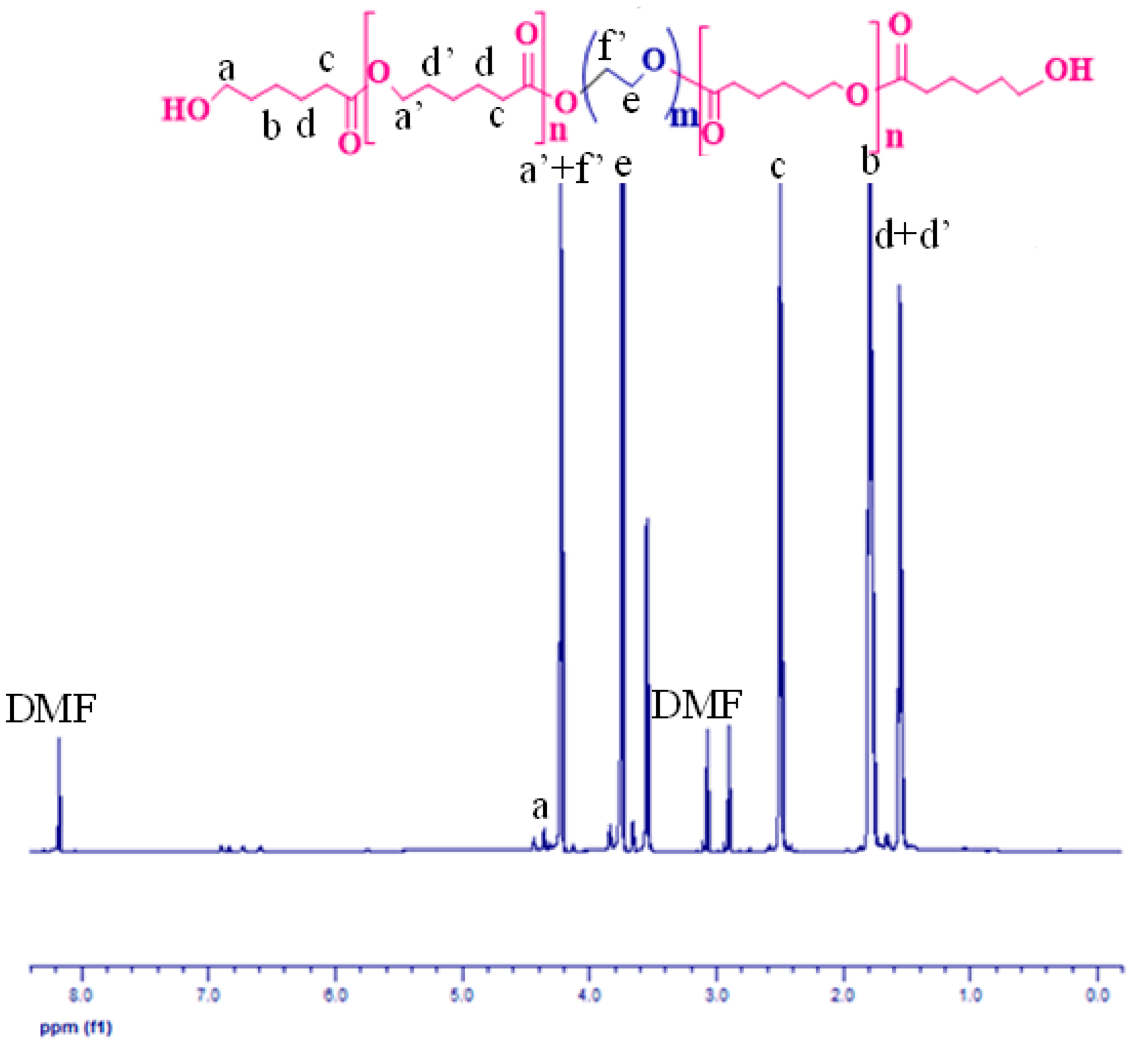

Figure S5. Structure characterization of synthesized copolymers by ^{1}H NMR for PCL-PEG dissolved in DMF as the solvent. The -CH2 proton of the PEG at 3.64 ppm (b) and the -CH_2OOC of PCL at 4.13 (a') confirms the formation of the copolymer of PCL-PEG.

Figure S6. Structure characterization of synthesized copolymers by ^{1}H NMR for PCL-PEG-PBC dissolved in DMF as solvent. The characteristic peaks of PBC benzene (g, h, i, k) and -CH (g', j) of a 6-member ring, confirm the formation of PCL-PEG-PBC.

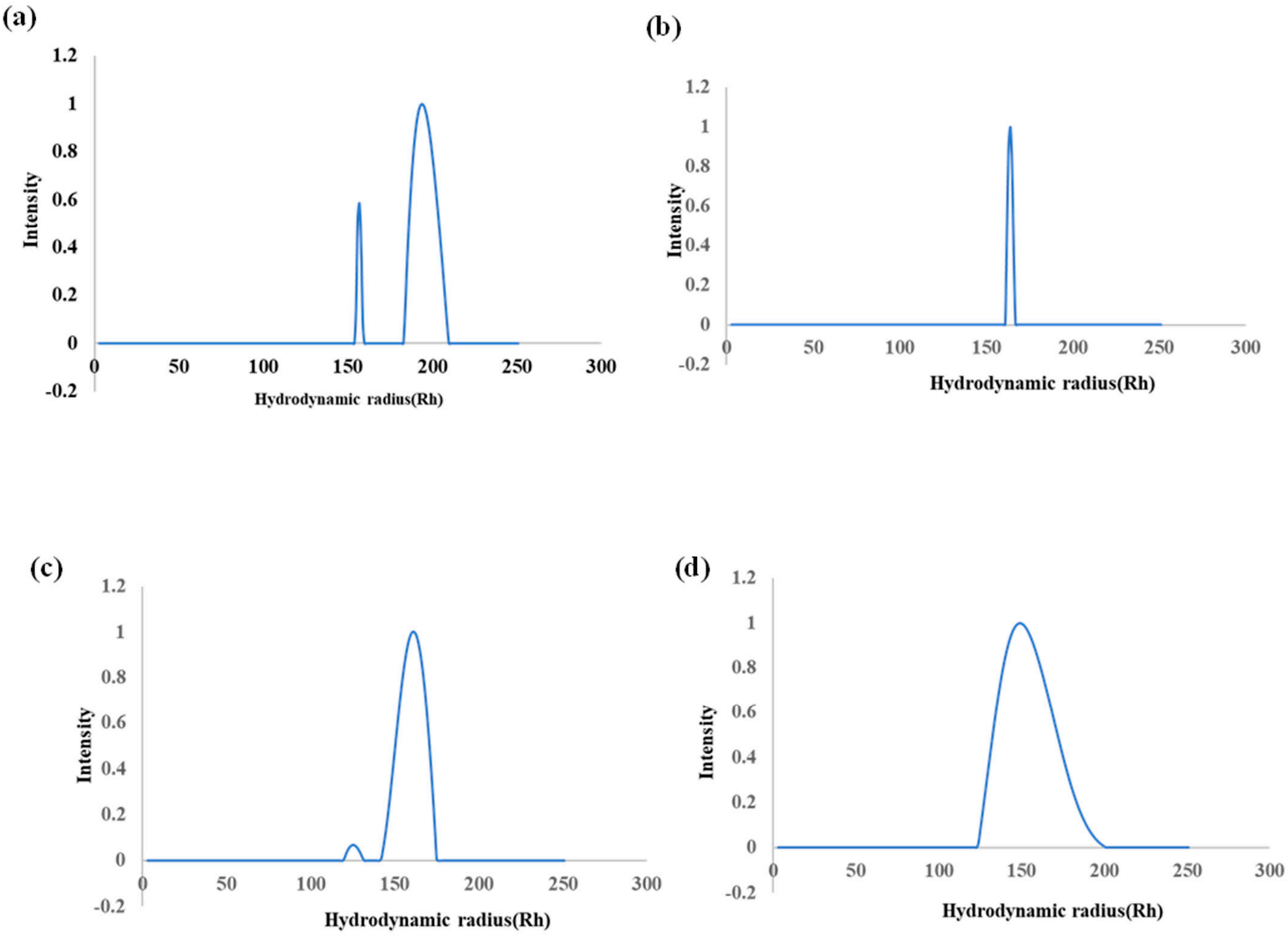

Figure S7. Particle size distribution images of (a) CA, (b) CA-PBC,(c) PCL-PEG, and (d) PCL-PEG-PBC.

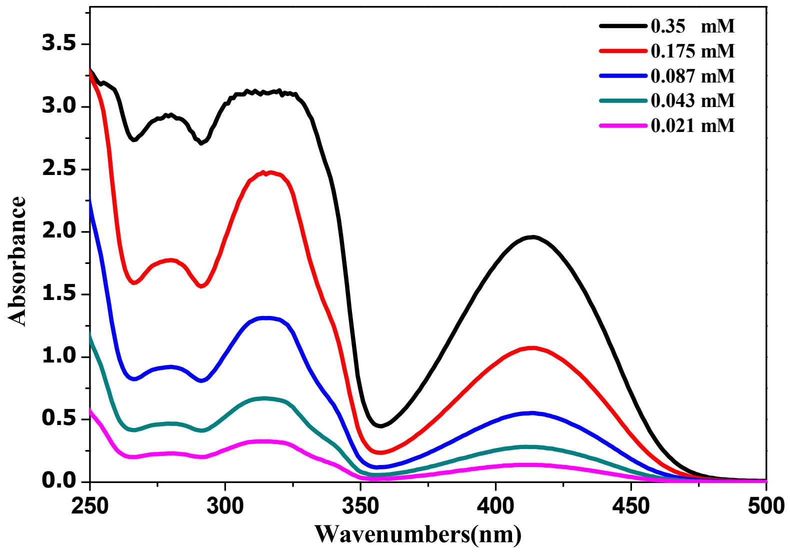

Figure S8. UV-Vis absorption spectra for PBC obtained from different concentrations(mM).

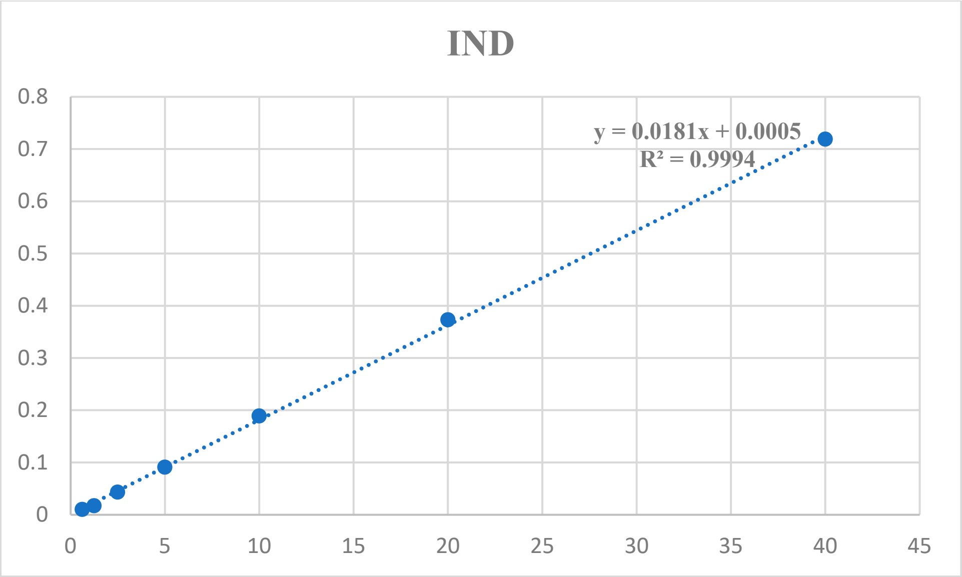

Figure S9. Standard curve for IND obtained from UV-Vis absorption spectra.

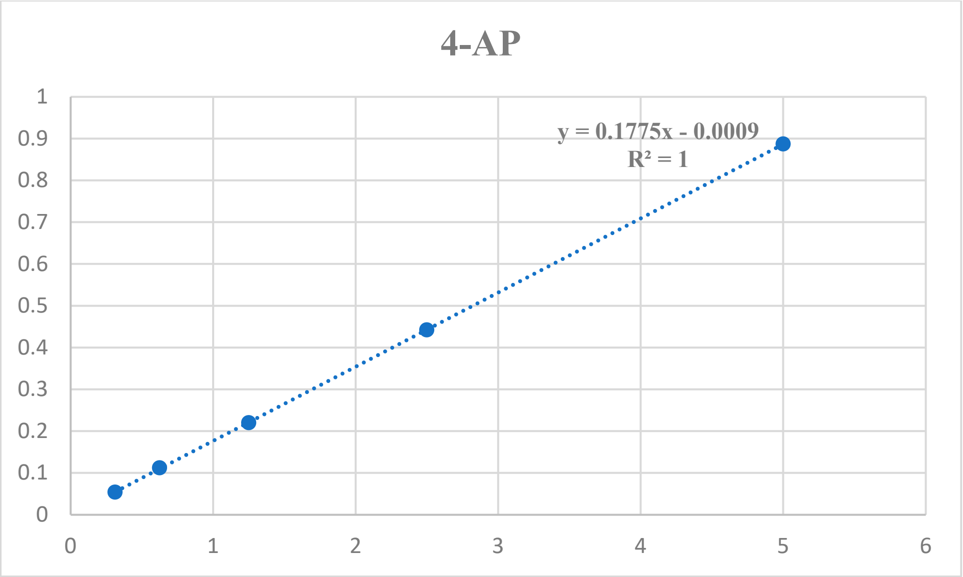

Figure S10. Standard curve for 4-AP obtained from UV-Vis absorption spectra.
